# The Probiotical Potential of Lactobacilli from Therapeutic Preventive Beverage Kurunga

**DOI:** 10.5195/cajgh.2014.176

**Published:** 2014-12-12

**Authors:** Lidia Stoyanova, Samat Kozhakhmetov, Almagul Kushugulova, Talgat Nurgozhin, Zhaxybay Zhumadilov, Andrey Bryukhanov, Maria Napalkova, Alexander Netrusov

**Affiliations:** 1 Department of Microbiology, Lomonosov Moscow State University, Russia; 2 Center for Life Sciences, Nazarbayev University, Astana, Kazakhstan

**Keywords:** kurunga, bacterial strain identification, L.diolivorans, probiotic

## Abstract

**Introduction:**

Kurunga is a dairy drink made of a mix of lactic acid and alcoholic fermentation, characterized by high biological value based on protein composition, amino acid spectrum, fatty acid composition of lipids, vitamin and mineral substances, and physiological activity of microbiota containing lactobacilli, lactococci, bifidobacteria, and yeast. Among the probiotic correctors of normal microbiota isolated from national products, lactobacilli was of particular interest, with regards to a therapeutic – preventive effect. The aim of the study was to examine the probiotic properties of lactobacilli from kurunga.

**Methods:**

We isolated lactic acid bacteria strains from kurunga. The isolated cultures were identified using common microbiological methods and phylogenetic analysis. The antibiotic activities of these strains were determined by measuring the growth inhibition zone of test cultures. The probiotic properties were measured as levels of resistance to bile and hydrochloric acids, in addition to the presence of superoxide dismutase (SOD) activity using the xanthine oxidase-cytochrome method. Proteolitic activity was determined at the various levels of pH (3.0, 4.2, 5.3, and 7.0).

**Results:**

According to the morphological, cultural, physiological, biochemical properties and the genotypic analysis of the oligonucleotides sequence of specific genes, the most effective strain was identified as Lactobacillus diolivorans KL-2 (GenBank database KC438372). The isolated strain suppressed the growth of Gram-positive bacteria, such as Bacillus, Staphylococcus, and Listeria sp., as well as Gram-negative bacteria, such as E.coli, Proteus, Salmonella sp. They also possessed fungicidal action (based on Penicillum, Aspergillus sp, and Candida sp.). The strain was resistant to the action of the bile acids at concentrations of 0.8% to 1.0% and hydrochloric acid. The strain KL-2 possessed a relatively high SOD activity (25.74 U/mg of protein), a low proteolytic activity at a pH 3.0 (4.74·10-3 PU/ml), and high proteolytic activity at pH 4.2 (294.74·10-3 PU/ml), pH 5.3 (330.52·10-3 PU/ml) and pH 7.0 (713.68·10-3 PU/ml).

**Conclusion:**

The unique properties of this strain, such as stability in the gastrointestinal tract, the wide spectrum of bactericidal and fungicidal action to the pathogenic species, the relatively high superoxide dismutase and proteolytic activities, and the absence of toxicity, make it a prime candidate for probiotic culturing.

